# Xenograft of Human Umbilical Mesenchymal Stem Cells from Wharton’s Jelly Differentiating into Osteocytes and Reducing Osteoclast Activity Reverses Osteoporosis in Ovariectomized Rats

**DOI:** 10.1177/0963689717750666

**Published:** 2018-03-22

**Authors:** Yu-Show Fu, Chia-Hui Lu, Kuo-An Chu, Chang-Ching Yeh, Tung-Lin Chiang, Tsui-Ling Ko, Mei-Miao Chiu, Cheng-Fong Chen

**Affiliations:** 1 Department of Anatomy and Cell Biology, School of Medicine, National Yang-Ming University, Taipei, Taiwan, Republic of China; 2 Department of Education and Research, Taipei City Hospital, Taipei, Taiwan, Republic of China; 3 Institute of Anatomy and Cell Biology, School of Medicine, National Yang-Ming University, Taipei, Taiwan, Republic of China; 4 Department of Internal Medicine, Division of Chest Medicine, Kaohsiung Veterans General Hospital, Taiwan, Republic of China; 5 Department of Obstetrics and Gynecology, Taipei Veterans General Hospital, Taipei, Taiwan, Republic of China; 6 Institute of Clinical Medicine, National Yang-Ming University, Taipei, Taiwan, Republic of China; 7 Department of Obstetrics and Gynecology, National Yang-Ming University, Taipei, Taiwan, Republic of China; 8 Taipei Municipal Jianguo High School, Taipei, Taiwan, Republic of China; 9 Department of Optometry, Shu-Zen College of Medicine and Management, Kaohsiung City, Taiwan, Republic of China; 10 Department of Medicine, Mackay Medical College, New Taipei, Taiwan, Republic of China; 11 Department of Orthopaedics and Traumatology, Division of Joint Reconstruction, Taipei Veterans General Hospital, Taipei, Taiwan, Republic of China; 12 Department of Orthopaedics, School of Medicine, National Yang-Ming University, Taipei, Taiwan, Republic of China; * These authors made equal contributions to this manuscript.

**Keywords:** umbilical cord-derived mesenchymal stem cells, Wharton’s jelly, xenograft, osteoporosis, osteoblast, osteoclast

## Abstract

We examined the effects of human umbilical cord-derived mesenchymal stem cells (HUMSCs) in Wharton’s jelly on ovariectomy (OVX)-induced osteoporosis by using in vitro and in vivo experiments. Two months after OVX, the rats gained weight and had a decreased serum estradiol level . Both micro-computed tomography (micro-CT) and histochemical analyses revealed a marked decrease in the bone volume (BV) and collagen content within the head, neck, and distal condyle of the femur, indicating that the osteoporosis animal model was successfully established 2 mo after bilateral OVX. Subsequently, 2.5 × 10^6^ HUMSCs were injected into the bone marrow cavity of the left femurs 2 mo after OVX. The rats were divided into the following groups: normal + phosphate-buffered saline (PBS), normal + HUMSCs, OVX + PBS, and OVX + HUMSCs. Two months after transplantation, both micro-CT imaging and histochemical staining revealed that the normal + HUMSCs group had higher BV and collagen content in the epiphysis and metaphysis than did the normal + PBS group. In the OVX + HUMSCs group, a substantial increase in the rod-shaped trabecular bone and the abundant accumulation of collagen were observed around the site of HUMSC transplantation. Plenty of transplanted HUMSCs remained viable and differentiated into osteoblasts. In addition, HUMSC transplantation reduced the number of osteoclasts. Compared with HUMSCs cultured alone, HUMSCs cocultured with osteoblasts showed that the percentage of cells differentiating into osteoblasts significantly increased. Furthermore, osteoclasts cocultured with HUMSCs had significantly decreased cellular activity and differentiation capability. HUMSC transplantation into the distal femur of OVX rats could locally stimulate osteocalcin synthesis, increase the trabecular bone, and inhibit osteoclast activity.

## Introduction

Osteoporosis is a common skeletal disease caused by increased bone turnover and decreased bone mass density, together with the deterioration of the bone microarchitecture, including reduced trabeculae and weaker bone microstructure. Therefore, a brittle skeleton increases the risk of bone fracture. In older people, bone fractures increase their immobility rate, reduce their quality of life, and render them incapable of self-care, increasing the burden on health-care systems.

Osteoporosis can be categorized as primary or secondary depending on its cause. Menopause-induced primary osteoporosis is caused by a rapid decline in the estrogen level, which results in higher activity of osteoclasts, the cells responsible for bone resorption, and in inactivity or a reduced number of osteoblasts, the cells responsible for bone formation. When the balance between bone resorption and formation is disturbed after menopause, the bone mass starts to decrease substantially, and the bone structure becomes less compact and easily breaks, ultimately leading to bone fracture^1^. Secondary osteoporosis is usually caused by other medical conditions that lead to an imbalance of bone resorption and formation. These medical conditions include genetic diseases (e.g., osteogenesis imperfecta), endocrine disorders (e.g., hyperthyroidism and hyperparathyroidism), gastrointestinal diseases (e.g., inflammatory bowel disease and chronic diarrhea), malnutrition or nutritional imbalance (e.g., vitamin D or calcium inadequacy), and drug abuse (e.g., steroid and excess alcohol consumption)^2–4^.

As bone resorption increases and bone formation decreases, this imbalance results in subsequent net bone loss, revealing thinner cortical bones, increased bone fragility, and decreased number, thickness, and separation of trabeculae within the bone marrow (BM) by the results of computed tomography (CT), X-ray examination, and histochemical staining. Many different types of medications can be clinically used for treating osteoporosis. Nevertheless, these medications exhibit adverse effects and limitations. Therefore, researchers are investigating the possibility of using stem cell therapy for osteoporosis.

In our laboratory, we have been exploring the therapeutic potential of mesenchymal stem cells (MSCs) in Wharton’s jelly from the umbilical cord for various diseases. Umbilical cords are waste materials after delivery. In contrast to embryos and BM, umbilical cords are relatively easy to obtain^5^. We successfully induced the differentiation of human umbilical cord-derived MSCs (HUMSCs) into neuronal cells in vitro^6^. In addition, these neuronal cells can be further differentiated into dopaminergic neurons. The transplantation of these dopaminergic neurons into the striatum of a parkinsonian rat could substantially mitigate its symptoms^7,8^. Moreover, HUMSCs were injected into the spinal cord, cerebral cortex, and hippocampus of rats for treating spinal cord injury, stroke, and epilepsy, respectively^9–11^. HUSMCs transplanted into different brain regions of rats could survive for at least 4 mo, indicating that HUMSCs do not induce an immune response or rejection in host animals. In addition to central nervous system diseases, the therapeutic potential of HUMSCs has been observed for diabetes, liver fibrosis, and peritoneal fibrosis in studies transplanting these cells into rats. These studies have reported that HUMSCs can survive in different organs of rats, suggesting that HUMSCs do not induce allograft or xenograft rejection and can serve as an excellent stem cell source^12–14^.

In this study, we used bilateral ovariectomized (OVX) rats, the most frequently used animal model, to mimic osteoporosis observed in menopausal women^15,16^. Two months after bilateral OVX, Sprague-Dawley (SD) rats were confirmed to have a prominent decrease in trabecular bone density by using micro-CT imaging and histochemical staining, and 2.5 × 10^6^ HUMSCs were then transplanted into the distal femurs of normal and OVX rats. The results showed that transplanted HUMSCs effectively mitigated the local severity of osteoporosis in rats.

## Materials and Methods

The use of human umbilical cords and laboratory animals in this study was approved by the Research Ethics Committee and the Animal Research Committee of the College of Medicine at National Yang-Ming University.

### Preparation of HUMSCs

Human umbilical cords were collected after delivery and stored in Hank’s balanced salt solution (HBSS; Biochrom L201-10) at 4 °C under sterile conditions. Umbilical cords were disinfected in 75% ethanol and placed in HBBS (Gibco 13185-052). Umbilical cords were cut vertically, and the blood vessels were removed. Subsequently, the mesenchymal tissue (Wharton’s jelly) was separated, diced into cubes of approximately 0.5 cm^3^, and centrifuged at 4,000 rpm for 5 min. After removal of the supernatant fraction, the precipitate was washed twice with serum-free Dulbecco’s modified Eagle’s medium (DMEM, Gibco 12100-046) and centrifuged at 4,500 rpm for 5 min. The mesenchymal tissue was subsequently treated with collagenase and trypsin. Fetal bovine serum (FBS; SH30071.03) was then added to neutralize the effect of trypsin. The harvested cells were considered HUMSCs. Finally, the cells were dispersed in DMEM containing 10% FBS and were counted for the following culture.

### Primary Culture of Rat Osteoblasts

Skulls were dissected from 1-day-old SD rats and cut into approximately 5 mm^2^ pieces by using sterile instruments, washed with sterile HBSS (Gibco 14185-052) to remove red blood cells, and digested with 0.1% trypsin and 0.1% collagenase type I, 3 times (15, 25, and 25 min) in a shaking water bath at 37 °C. DMEM with 10% FBS was then added to inactivate trypsin, followed by centrifugation at 4,000 rpm for 5 min, and the cells were resuspended in DMEM with 10% FBS. The cells were then counted and cultured. After identification through alkaline phosphatase staining and core-binding factor alpha 1 (Cbfa-1) polymerase chain reaction (PCR), the cells were cultured in a 10-cm culture dish; after reaching confluence in approximately 6 to 7 d, the cells were subcultured. Osteoblasts at the second passage were used in the study.

### Primary Culture of Rat Osteoclasts

After shaving and disinfection with ethanol, both the femurs of rat hind limbs were removed. In a laminar flow hood, the femurs were cut at both ends, and the contents of the marrow were flushed with 10% FBS α-minimum essential medium (MEM) (Gibco A10490-01) by using a syringe with a 20-gauge needle. The contents were centrifuged at 1,500 rpm for 5 min. The supernatant fraction was discarded, and the precipitate was washed with α-MEM containing 10% FBS and centrifuged at 1,500 rpm for 5 min. After removal of the supernatant, the precipitate was resuspended in 10 cc α-MEM containing 10% FBS and seeded in a 10-cm culture dish. After overnight culture, the culture dish was rocked gently to suspend the osteoclast progenitor cells. The supernatant was centrifuged at 1,500 rpm for 5 min. After removal of the supernatant, the cells were resuspended in α-MEM with 10% FBS and cultured in 25 ng/mL macrophage colony stimulating factor (M-CSF) (PeproTech 315-02). The differentiation of osteoclasts from osteoclast progenitor cells was induced by treatment with 10 or 100 ng receptor activator of nuclear factor kappa-B ligand (RANKL) (PeproTech 315-11) for 9 d^17^.

### Assay of Osteoblast Differentiation in Osteoblasts Cultured Alone or Osteoblast and HUMSC Cocultures Using Alkaline Phosphatase Staining

To explore the interaction between HUMSCs and osteoblasts, osteoblasts were cultured alone or with HUMSCs in a Transwell System. The coculture system consisted of upper and lower chambers separated by a distance not physically traversable by the cells. However, the chambers shared the same medium, which covered both cultures, thus allowing access to both cultures through humoral factors. A porous membrane with multiple pores of 8 μm in diameter comprised the bottom of the upper chamber, which allowed only the movement of the medium in the membrane but not the actual mixing of the cells. Osteoblasts (2 × 10^5^ cells) were cultured alone or cocultured with HUMSCs using DMEM containing 2% bovine serum albumin (BSA). As an in vitro coculture system, osteoblasts were seeded onto 6-well plates, and 10^5^ HUMSCs were cultured on Transwells with a pore diameter of 8 μm. Transwells seeded with HUMSCs were placed on plates with osteoblasts. After 24 h, 48 h, or 72 h, the levels of cell differentiation were compared between single-culture and coculture systems. Subsequently, ddH_2_O with a dissolved 5-bromo-4-chloro-3-indolyl phosphate/nitro blue terazolium (Sigma-Aldrich B5655) tablet was added into the culture dish, kept away from light, and incubated in an oven at 37 °C. After 1 h, the alkaline expression level was observed.

### Assay of HUMSCs Differentiating into Osteocytes in HUMSCs Cultured Alone or HUMSC and Osteoblast Cocultures Using Alkaline Phosphatase Staining

HUMSCs cultured alone or with osteoblasts were cultured in osteoblastic differentiation medium containing 10% FBS, 10^−8^ M dexamethasone (Sigma-Aldrich D4902), 50 ng/mL l-ascorbic acid 2-phosphate (Sigma-Aldrich A8960), and 10 mM β-glycerophosphate (Sigma-Aldrich G9422) in DMEM low glucose (Gibco 11885092). The medium was changed twice per week. After 2-wk culture, the alkaline phosphatase expression level in HUMSCs was examined.

### Number of Osteoclasts Examined Using Tartrate-resistant Acid Phosphatase Staining

To explore the interaction between HUMSCs and osteoclasts, osteoclasts were cultured alone or with HUMSCs in a Transwell System. Osteoclasts were treated with 10 or 100 ng RANKL (PeproTech 315-11) for 9 d to induce differentiation into osteoclasts. Acid phosphatase leukocyte (Sigma-Aldrich 387A) was added and incubated at 37 °C for 1 h in an oven protected from light. Osteoclasts that were stained purple and had a nucleus number of three or above were counted.

### Assessment of Osteoclast Activity Using Osteoassay

Osteoclasts that were cultured in the presence or absence of HUMSCs were seeded onto a 24-well osteoassay plate (Corning Inc., Corning, NY, USA 3987), a plate coated with inorganic crystalline calcium phosphate, and were treated with 10 or 100 ng RANKL for 9 d to induce differentiation. The cells were then dissolved with a bleach solution (6% NaOCl and 5.2% NaCl), washed with ddH_2_O, and photographed. The resorption area was quantified and represented the osteoclast activity.

### Experimental Animals

Animals were housed in transparent plastic cages (45 × 24 × 20 cm^3^) under a 12-/12-h reversed light–dark cycle. Lights were switched on at 07:30, and the temperature was kept constant (25 ± 2 °C). The animals were provided standard rodent chow (laboratory animal diet MF8) and drinking water ad libitum. The frequency of bedding change was once per week.

### Animal Model of Bilateral OVX-induced Osteoporosis

Female SD rats were anesthetized, and their abdomens (close to the hip joints) were shaved bilaterally. The skin was disinfected with iodine-immersed cotton swabs.

Incisions were made through the skin and the muscle wall. Subsequently, the ovaries were pulled away from the incision site gently by using forceps, ligated with 4-0 nylon sutures, and removed. The muscle and skin layers were then sutured separately.

### Animal Grouping

Animals were divided into 4 groups as follows:

1. Normal + PBS group: normal SD rats did not receive OVX but underwent sham surgery (opening of the abdominal cavity) at the age of 1 mo. PBS was administered into the distal femurs at the age of 3 mo.
2. Normal + HUMSCs group: normal SD rats did not receive OVX. At the age of 3 mo, 2.5 × 10^6^ HUMSCs were transplanted into the left distal femur.
3. OVX + PBS group: SD rats received bilateral OVX at the age of 1 mo. PBS was administered into the distal femurs at the age of 3 mo.
4. OVX + HUMSCs group: SD rats received bilateral OVX at the age of 1 mo. At the age of 3 mo, 2.5 × 10^6^ HUMSCs were transplanted into the left distal femur.

### Transplantation of HUMSCs

HUMSCs were treated with 0.05% trypsin (Biochrom L2153) for 4 min, washed with DMEM containing 10% FBS, and centrifuged at 1,500 rpm for 5 min. After removal of the supernatant fraction, 2.5 × 10^6^ HUMSCs were resuspended in 0.1 mL of 0.1 M sterile PBS. Two months after OVX, rats were anesthetized, and 0.1 mL of HUMSC suspension was injected using a 22-gauge needle. Before the injection of HUMSCs, the needle was inserted 8 mm into the BM cavity of the left femur through the distal condyle.

### Tissue Preparation

Rat femurs were obtained, placed in a fixation solution for 24 h, and decalcified in 20% ethylenediaminetetraacetic acid (EDTA) for 3 to 4 wk (20% EDTA in ddH_2_O was changed twice every week). The decalcified tissue was washed with tap water for 8 h and immersed in 0.1 M PB. The tissue was dehydrated by passing it through a series of increasing alcohol concentrations (70%, 80%, 95% twice, and 100% 6 times). The tissues were then submerged in a series of graded xylene–paraffin baths (xylene, xylene: paraffin [1:1], xylene: paraffin [1:3], and pure paraffin) and were finally embedded in paraffin. A series of consecutive 7-μm-thick sagittal sections were obtained using a microtome. Approximately 250 sections could be collected from one femur. The sections were placed on slides for various staining (the allocation scheme of femoral histologic sections for staining is illustrated in Supplemental Figs. 1 and 2).

**Fig. 1.**
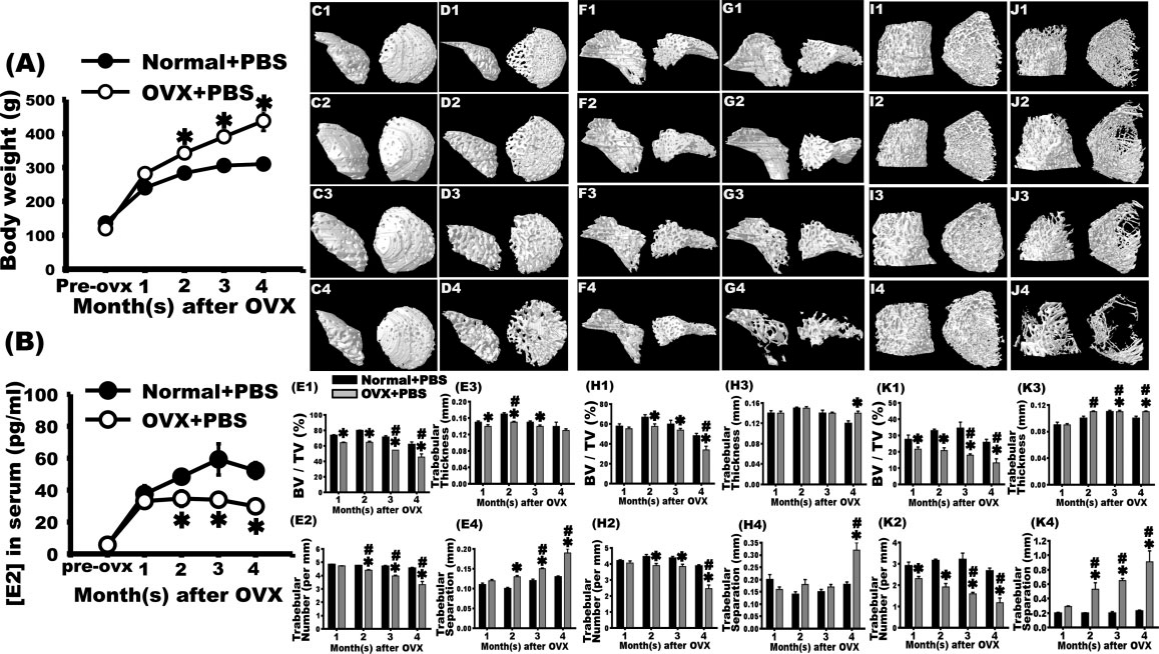
Bilateral ovariectomy (OVX) induced osteoporosis in rats, observed as a decrease in the femoral bone mass through micro-computed tomography (CT). Two months after surgery, the weight of rats in the OVX + phosphate-buffered saline (PBS) group increased significantly compared with that of rats in the normal + PBS group at the same age. This difference was observed until the fourth month after surgery. The OVX + PBS group had decreased estradiol levels in the second month after OVX. In contrast to the normal + PBS group at the same age, the serum estradiol level markedly declined, and this decrease was observed until the fourth month after OVX. Micro-CT was used to observe trabecular alterations within the left femoral head (C1–C4, D1–D4), neck (F1–F4, G1–G4), and distal condyle (I1–I4, J1–J4). Dense arrangements of trabeculae were observed in the normal + PBS group 1 (C1, F1, and I1), 2 (C2, F2, and I2), 3 (C3, F3, and I3), and 4 (C4, F4, and I4) months after sham surgery. In the first month after bilateral OVX, slight changes in bone mass were observed (D1, G1, and J1). In the second month, osteoporotic trabeculae were observed (D2, G2, and J2). In the third (D3, G3, and J3) and fourth (D4, G4, and J4) months after OVX, severe osteoporotic loss of bone mass was observed. The bone volume fractions (E1, H1, and K1), trabecular number (E2, H2, and K2), trabecular thickness (E3, H3, and K3), and trabecular separation (E4, H4, and K4) were quantified at the femoral head (E1–E4), neck (H1–H4), and distal condyle (K1–K4). The results revealed that these parameters decreased significantly in the second month after OVX. These trends were observed until the third month. In the fourth month after OVX, these parameters were even higher. *OVX + PBS group versus normal + PBS group at the same time point, *P* < 0.05. ^#^Versus OVX + PBS group 1 month after OVX, *P* < 0.05.

**Fig. 2.**
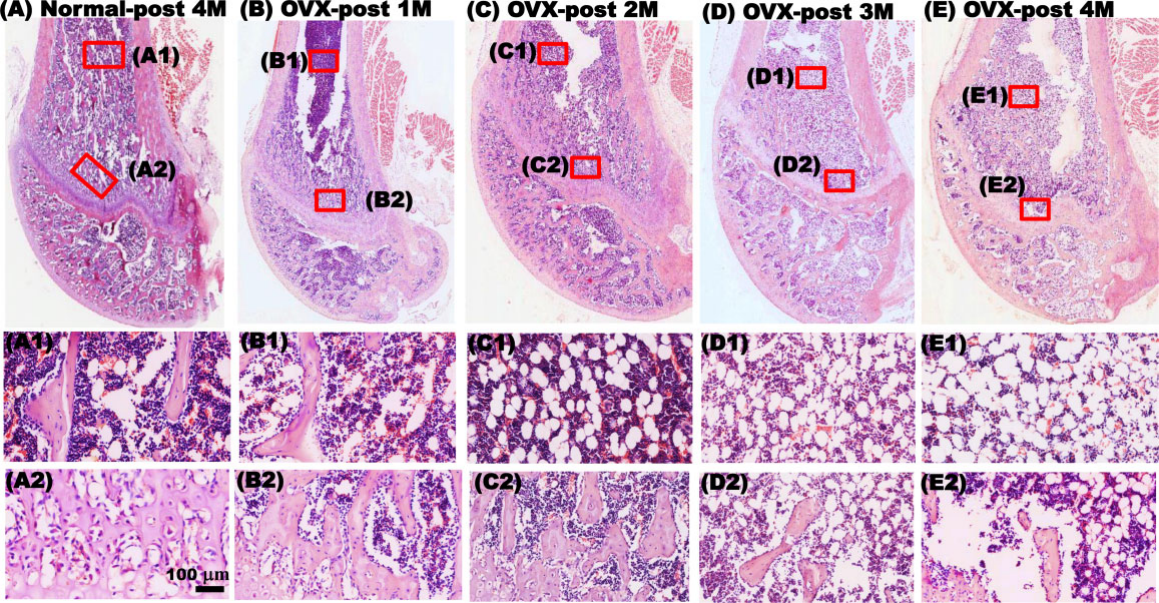
Hematoxylin and eosin (H&E) staining demonstrated that bilateral ovariectomy (OVX) results in osteoporosis with loss of bone mass at the distal femur condyle of rats. H&E staining revealed that the trabeculae showed compact arrangements with little interspaces in the epiphysis and metaphysis of the distal femoral in the normal + phosphate-buffered saline (PBS) group 4 mo after surgery (A and A2). The trabeculae and few adipocytes were observed in the bone marrow of the femoral shaft (A1). One month after OVX, the OVX + PBS group showed organized arrangement and slightly increased interspaces of trabeculae in the epiphysis (B and B2). Some trabeculae and adipocytes were still present in the bone marrow of the diaphysis (B1). Remarkably decreased trabeculae with a loosely arranged structure were observed in the metaphysis in the second, third, and fourth months after OVX (C, C2, D, D2, E, and E2). A tremendous amount of adipocytes replaced the trabeculae in the metaphysis, and the trabeculae could barely be observed at these time points (C1, D1, and E1). Scale bar: 100 μm.

### Hematoxylin and Eosin (H&E) Staining

After dehydration, the tissue sections were immersed in hematoxylin for 5 min, washed with tap water for 15 min, quick dipped in 1% acid alcohol, washed with tap water for 15 min, immersed in 70% ethanol for 3 min, immersed in eosin for 1 min, washed with tap water for 15 min, and dehydrated by immersing in a series of increasing concentrations of alcohol (95% twice and 100% twice; 2 min each). The sections were then immersed in xylene twice for 5 min each, mounted with Permount, and photographed using an optical microscope. For every femur, eight intact tissue sections were used for quantification. The percentages of the area occupied by the trabeculae in the metaphysis, 1 mm under the epiphyseal plate of the distal femoral condyle, were quantified.

### Sirius Red Staining

After dehydration, the tissue sections were immersed in 0.1% Sirius red (in picric acid; Sigma-Aldrich 365548) for 14 min, dehydrated by immersing in a series of increasing concentrations of alcohol (50%, 70%, 80% 90%, 95%, and 100% twice) for 30 s each, immersed in xylene twice for 5 min each, mounted using a mounting medium, and observed and photographed using an optical microscope. For every femur, eight intact tissue sections were used for quantification. Tissues containing collagen were stained in red. The percentages of area occupied by the trabeculae in the metaphysis, 1 mm under the epiphyseal plate of the distal femoral condyle, were quantified.

### Goldner’s Trichrome Staining

According to Goldner’s trichrome method (EMS 26386), tissue sections were placed in a mixture of hematoxylin A and hematoxylin B for 10 min, immersed in 0.1% acetate, destained in Orange G, submerged in 0.1% acetate for 30 s, stained in light green G solution for 10 min, immersed in 0.1% acetate for 5 min, and dehydrated by immersing them in a series of increasing alcohol concentrations (95% twice and 100% twice; 5 min each). Sections were then immersed in xylene twice for 5 min each, mounted using a mounting medium, and observed and photographed using an optical microscope. Nuclei were stained in black, whereas red blood cells and muscles were stained in red. Tissues containing collagen were stained in green, such as the trabecular bone, tendons, and ligaments. Eight intact tissue sections were used for quantification for every femur. The percentages of area occupied by the trabeculae in the metaphysis, 1 mm under epiphyseal plate of the distal femoral condyle, were quantified.

### Tartrate-resistant Acid Phosphatase Staining

After dehydration, tissue sections were submerged in acid phosphatase leukocyte (Sigma-Aldrich 387A) at 37 °C for 60 min, immersed in hematoxylin for 2 min for nuclei staining, washed with tap water for 5 min, mounted using a mounting medium, and observed and photographed using an optical microscope for quantification. Osteoclasts were the cells with multiple nuclei and stained purple. For every femur, 6 intact tissue sections were used for quantification. The number of purple osteoclasts surrounding the trabeculae in the area of 1 mm under the epiphyseal plate of the distal femoral condyle was quantified. The osteoclast index was calculated as the number of osteoclasts divided by the trabecular bone area (mm^2^).

### Micro-CT

In the present study, femurs were scanned ex vivo by using the micro-CT equipment (SkyScan 1076) of Taiwan Mouse Clinic to analyze the head, neck, and distal condyle of femurs. The data obtained for the femoral head and femoral distal condyle were areas located 8 mm from the distal end to the diaphysis.

The bone volume/total volume (BV/TV) is the relative BV or BV fraction and is expressed in percentage. The BV/TV decreases during osteoporosis.

The trabecular thickness (Tb.Th) represents the mean thickness of the trabeculae and is expressed in millimeters. The Tb.Th decreases during osteoporosis.

The trabecular number (Tb.N) is the number of trabeculae measured by counting the number of intercepts of the bone and background tissues in a given length and is expressed in 1 per mm. The Tb.N reduces during osteoporosis.

The trabecular separation (Tb.Sp) is the spacing between the trabecular bone and represents the average width of the marrow among the trabecular bone. It is expressed in millimeters. An increase in the Tb.Sp indicates an increase in bone resorption and may suggest osteoporosis.

### Reverse Transcription Polymerase Chain Reaction for Detecting Human Osteocalcin in the Femur of the Rats

Total RNA was freshly isolated from the rat femur using the TRIzol Reagent (Invitrogen, USA) and reverse transcribed into complementary DNA with SuperScript III (18,080, Invitrogen). Specific primers for PCR were designed to detect only human osteocalcin with primer express software (NCBI, Primer-BLAST, National Institutes of Health (NIH), Bethesda, MD, USA). PCR products were amplified with 40 cycles of 94 °C for 30 s, 58 °C for 30 s, and 72 °C for 30 s and resolved on a 2% agarose gel. Human osteosarcoma cell line was used as a positive control.

The primer sequences were as follows:

Human osteocalcin:
Sense: 5′-CTCACACTCCTCGCCCTATT-3′
Antisense: 5′-TCAGCCAACTCGTCACAGTC-3′ 245 bp
Human glyceraldehyde 3-phosphate dehydrogenase (GAPDH):
Sense: 5′-TTCCACCCATGGCAAATTCCATGG-3′
Antisense: 5 5′-GGTCAGGTCCACCACTGACACG-3′ 589 bp
Rat GAPDH:
Sense: 5′-CTCTACCCACGGCAAGTTCAAC-3′
Antisense: 5′-GGTGAAGACGCCAGTAGACTCCA-3′ 160 bp

### Double Staining of Antihuman-Specific Nuclear Antigen and Anti-Osterix

To assess the differentiation of implanted HUMSCs, double immunostaining was performed with human-specific nuclear antigen and anti-osterix to localize osteoblasts differentiated from HUMSCs. Femur sections (7 μm) were fixed with 4% paraformaldehyde (PFA) in a 0.1 M phosphate buffer. The sections were then reacted with mouse antihuman-specific nuclear antigen antibody (1:100, MAB1281, United Chemicon, Rosemont, IL, USA) and anti-osterix (1:100, ab94744, Abcam) at 4 °C for 18 h, washed with 0.1 M PBS, and reacted with rhodamine-conjugated goat anti-mouse IgG and fluorescein-conjugated goat anti-rabbit IgG at room temperature for 1 h.

### Statistical Analyses

One- or two-way analysis of variance (ANOVA) was used to compare all means, followed by least significant difference posteriori tests. All data are presented as means ± standard error of the mean (SEM). A statistically significant difference was defined as *P* < 0.05.

## Results

### Body Weight and Estradiol Level Changed after OVX

The weight of rats in the normal + PBS group increased from 137.76 ± 9.8 to 310.00 ± 14.1 g at 1, 2, 3, and 4 mo after sham surgery. By contrast, the average weight of rats in the OVX + PBS group increased from 126.70 ± 8.27 g to 437.00 ± 29.38 g. Compared with rats in the normal + PBS group, the weight of rats in the OVX + PBS group significantly increased 2 mo after OVX, and this significance remained until 4 mo after OVX (Fig. 1A).

The analysis of the serum estradiol level showed that 1- and 2- to 5-mo-old female rats had serum estradiol levels of 5.70 ± 1.24 pg/mL and 37.49 ± 5.24 to 52.22 ± 5.01 pg/mL, respectively. By contrast, those in the OVX + PBS group had serum estradiol levels of 33.10 ± 2.76 to 29.79 ± 1.16 pg/mL in the first to fourth months after OVX. Starting from the second month after OVX, the serum estradiol level in the OVX + PBS group significantly decreased compared with that in the normal + PBS group at the same age. Such a trend was observed until the fourth month after OVX (Fig. 1B).

### Micro-CT Analysis Revealed Decreased Trabeculae in the Head, Neck, and Distal Condyle of the Left Femur after OVX

To quantify trabecular alterations, micro-CT was used to examine the head, neck, and distal condyle of the femur 1, 2, 3, and 4 mo after bilateral OVX.

The left femurs of rats in the normal + PBS group showed dense trabecular 1 to 4 mo after sham surgery (Fig. 1C1–C4). A slight decrease in the trabeculae was observed 1 mo after OVX (Fig. 1D1). The trabeculae decreased prominently 2 mo after OVX (Fig. 1D2). In the third and fourth months after OVX, the Tb.Sp increased and the severity of osteoporosis worsened with time (Fig. 1D3–D4). Micro-CT analysis revealed that compared with rats in the normal + PBS group, the BV fraction (BV/TV), Tb.N, and Tb.Th significantly decreased in the left femurs of rats in the OVX + PBS group (*P* < 0.05, Fig. 1E1–E3). Moreover, the Tb.Sp significantly increased in the OVX + PBS group compared with the normal + PBS group (*P* < 0.05, Fig. 1E4).

From the first to fourth months after sham surgery, the trabeculae within the femoral neck and distal femoral condyle displayed dense patterns in the normal + PBS group (Fig. 1F1–F4, 1I1–I4). No significant alteration was observed in the OVX + PBS group in the first month after OVX (Fig. 1G1, J1). In the second month after OVX, the trabeculae slightly decreased (Fig. 1G2, J2). In the third and fourth months after OVX, the trabeculae markedly decreased (*P* < 0.05, Fig. 1G3–G4, 1J3–J4). Micro-CT analysis revealed that compared with the normal + PBS group, the BV fraction, Tb.N, and Tb.Th substantially decreased in the OVX + PBS group (*P* < 0.05, Fig. 1H1–1H3, 1K1–K3). A significant increase in Tb.Sp was also observed (*P* < 0.05, Fig. 1H4, 1K4).

According to the micro-CT data, osteoporosis was observed in the head, neck, and distal condyle of the femurs of rats in the OVX + PBS group.

### H&E Staining Revealed Significantly Decreased Trabeculae in the Metaphysis of the Distal Femoral Condyle and Increased Adipocytes within the BM Cavity after OVX

The H&E staining results showed that from the first to fourth months after sham surgery, the trabeculae in the metaphysis of the distal femoral condyle displayed compact arrangement, and the Tb.Sp was small (Fig. 2A, A2). Only few adipocytes were present, and observable trabeculae were still distributed within the BM cavity (Fig. 2A1). In the OVX + PBS group, the trabeculae in the metaphysis of the distal femoral condyle slightly decreased in the first month after OVX (Fig. 2B, B2). Some adipocytes were present in the BM (Fig. 2B1). In the second month after OVX, the trabeculae in the metaphysis of the distal femoral condyle decreased significantly, and the trabeculae separation extended (Fig. 2C, C2). The number of adipocytes residing in the BM increased (Fig. 2C1). In addition, the phenomena of decreased trabeculae and increased adipocytes was observed until the third (Fig. 2D-D2) and fourth months after OVX (Fig. 2E-E2).

### Goldner’s Trichrome Staining Showed Decreased Collagen in the Trabecular Bone in the Metaphysis of the Distal Femoral Condyle after OVX

**Fig. 3.**
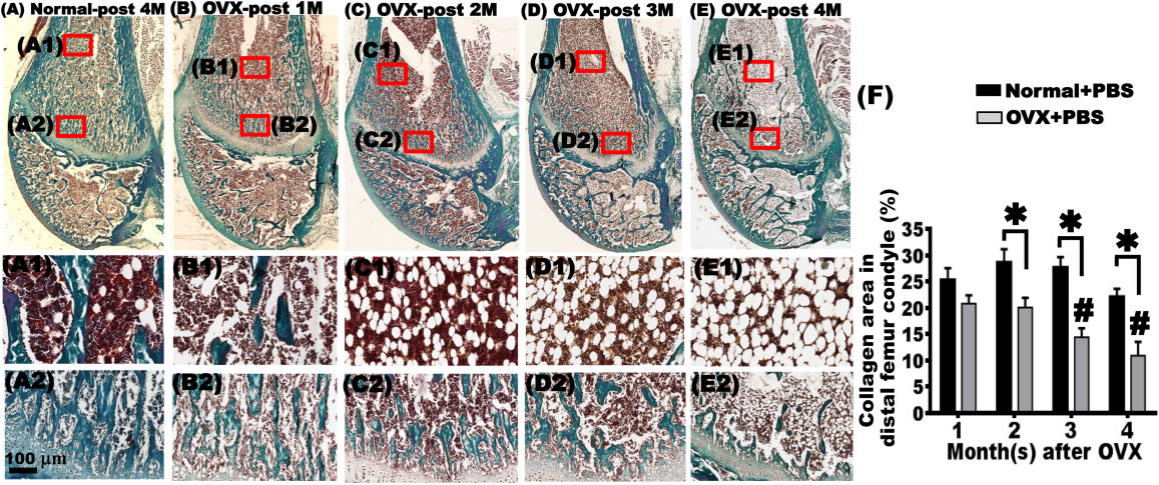
Goldner’s trichrome staining showed that bilateral ovariectomy (OVX) in rats reduced the collagen content at the distal femur. Goldner’s trichrome staining revealed organized arrangements with little interspaces in the trabeculae in the metaphysis of the distal femoral condyle in the normal + phosphate-buffered saline (PBS) group 4 mo after surgery (A and A2). The trabeculae and few adipocytes were still observed in the bone marrow of the femoral shaft (A1). In the first month after OVX, the OVX + PBS group exhibited trabeculae with organized arrangement and elevated interspaces in the metaphysis (B and B2). Some trabeculae and adipocytes were still observed in the bone marrow of the diaphysis (B1). Considerably decreased trabeculae in the metaphysis with a loosely arranged structure were observed in the second, third, and fourth months after OVX (C, C2, D, D2, E, and E2). The trabeculae in the metaphysis were replaced by a substantial amount of adipocytes in the second, third, and fourth months after OVX in the bone marrow (C1, D1, and E1). Compared with the first month after OVX, the aggravation of the femur trabecular bone area (%) increased with time (F). Scale bar: 100 μm. *OVX + PBS group versus normal + PBS group at the same time point, *P* < 0.05. ^#^ versus OVX + PBS group 1 mo after OVX, *P* < 0.05.

Goldner’s trichrome staining stains collagen in green in the trabecular bones, tendons, and ligaments. From the first to fourth months after sham surgery, the normal + PBS group had dense and organized collagen. Limited interspaces within collagen were present in the metaphysis (Fig. 3A, A2). Many blood cells stained in brown were observed in the BM cavity, with some white interspaces reaming, where adipocytes might have resided. The trabeculae were found to be distributed in the BM cavity (Fig. 3A1). In the OVX + PBS group, thinner and lower content of collagen was found in the metaphysis of the distal femoral condyle in the first month after OVX (Fig. 3B, B2). An increased number of adipocytes was also found in the BM cavity (Fig. 3B1). In the second month after OVX, collagen was sparse and short with increased interspaces (Fig. 3C, C2). The number of adipocytes also increased with reduction in the trabeculae in the BM cavity (Fig. 3C1). In the third and fourth months after surgery, the trabeculae in the metaphysis showed a looser structure and larger interspaces. The BM was filled with adipocytes, and fewer blood cells were present (Figs. 3D-D2, 3E-E2).

In the normal + PBS group, the quantification of the collagen area stained green in the metaphysis of the distal femur ranged from 42.99% ± 1.96% to 56.30% ± 2.47% from the first to fourth months after sham surgery. From the second month after OVX, the OVX + PBS group showed a substantial decrease in the collagen area compared with the normal + PBS group at the same age. This trend was observed until the third and fourth months after OVX. Compared with the first month, the collagen loss was significant in third and fourth months (*P* < 0.05, Fig. 3F).

### Sirius Red Staining Indicated a Significant Decrease in the Collagen of the Trabecular Bone in the Metaphysis of the Distal Femoral Condyle after OVX

Sirius red staining stains collagen in red in the trabecular bones, tendons, and ligaments. From the first to fourth months after surgery, collagen in the metaphysis of the distal femoral condyle was closer to the epiphyseal plate, denser, more organized, and had a more compact mesh-like structure (Supplemental Fig. 3A1–A4). No statistical difference was found from the first to fourth months after sham surgery. Nevertheless, a looser structure and increased perforated collagen were observed as the distance from the epiphyseal plate increased (Supplemental Fig. 3A1–A4). In the first month after OVX, collagen in the metaphysis of the distal femoral condyle slightly decreased (Supplemental Fig. 3B1). Collagen decreased significantly 2 mo after OVX (Supplemental Fig. 3B2, 3C). In the third and fourth months after OVX, a marked decrease in collagen was observed. The irregular mesh-like structure of red collagen was observed in areas closer to the epiphyseal plate (Supplemental Fig. 3B3–B4, 3C).

### Micro-CT Analysis Indicated that Transplanted HUMSCs Promoted Osteogenesis in the Distal Femoral Condyle in the Normal + PBS Group

**Fig. 4.**
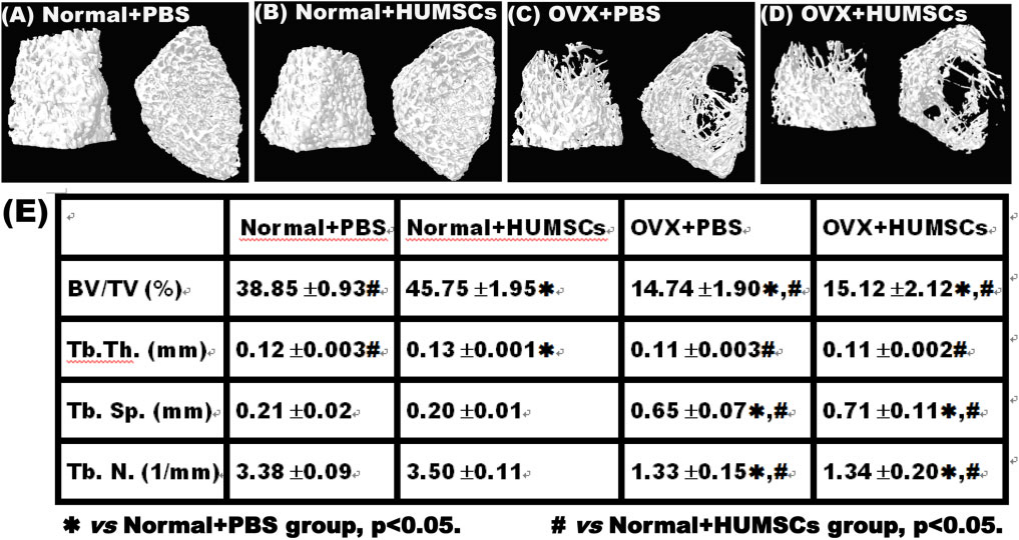
Micro-computed tomography (CT) 3-D imaging revealed that human umbilical mesenchymal stem cell (HUMSC) transplantation into the bone marrow of the normal rat femur increased bone mass in the distal condyle. Using micro-CT imaging, trabecular alterations in the left distal femoral condyle were estimated in the normal + phosphate-buffered saline (PBS; A), normal + HUMSCs (B), ovariectomy (OVX) + PBS (C), and OVX + HUMSCs (D) groups. The bone volume fractions (BV/TV), trabecular thickness (Tb.Th), and trabecular number (Tb.N) were significantly lower in the OVX + PBS and OVX + HUMSCs groups than in the normal + PBS and normal + HUMSCs groups (E). The BV/TV, Tb.Th, and Tb.N in the normal + HUMSCs group were significantly higher than those in the normal + PBS group (E). *Versus normal + PBS group, *P* < 0.05. ^#^Versus normal + HUMSCs group, *P* < 0.05.

Micro-CT analysis showed compact orientation of the trabecular bone in both the left and right distal femoral condyles in the normal + PBS and normal + HUMSCs groups (Fig. 4A, 4B). However, the percentages of BV/TV and Tb.Th of the left distal femoral condyle were significantly higher in the normal + HUMSCs group than in the normal + PBS group (Fig. 4E). In the OVX + PBS and OVX + HUMSCs groups, decreased and looser trabeculae were observed in the distal femoral condyles (Fig. 4C, 4D). Compared with the normal + PBS and normal + HUMSCs groups, the OVX + PBS and OVX + HUMSCs groups showed significant decreases in the percentages of BV/TV, Tb.Th, and Tb.N and a significant increase in Tb.Sp (Fig. 4E).

### Transplantation of HUMSCs into the Rat BM Cavity Increased Local Osteogenesis in OVX Rats

**Fig. 5.**
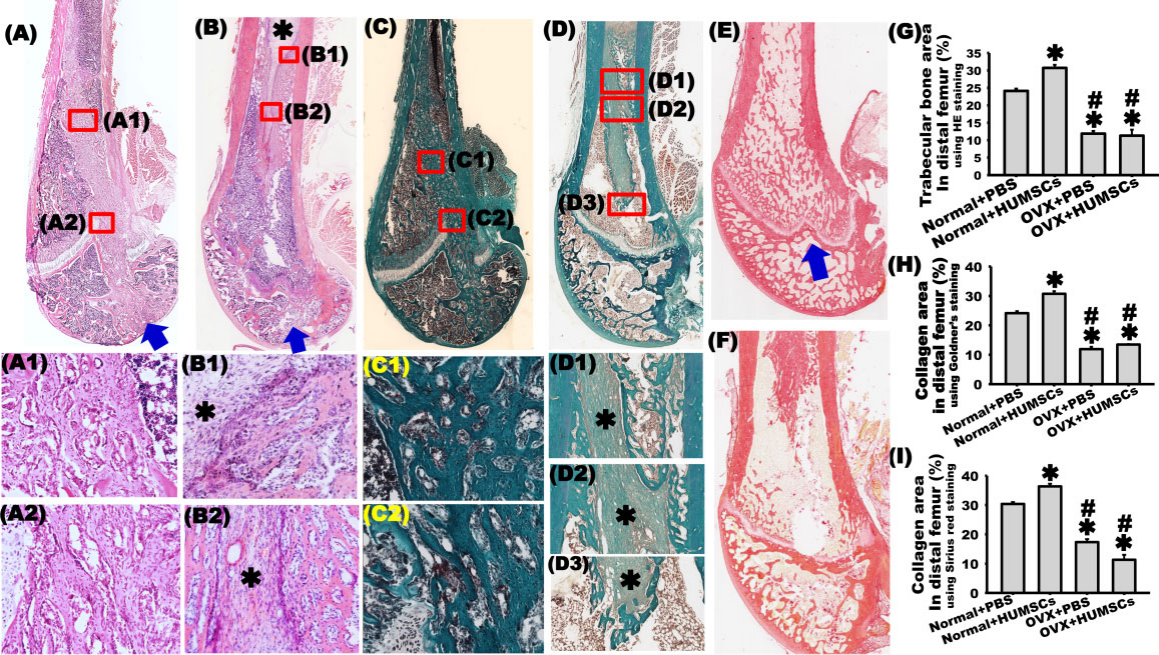
Transplantation of human umbilical cord-derived mesenchymal stem cells (HUMSCs) locally promoted osteogenesis in rats with osteoporosis. Hematoxylin and eosin (H&E) staining was applied 2 mo after transplantation of HUMSCs into the left distal femur in normal + HUMSCs and ovariectomy (OVX) + HUMSCs groups to evaluate trabecular alterations (A, B). The results showed dense arrangements of the trabeculae in the epiphysis and metaphysis of the distal femoral (A, A2) and in the bone marrow cavity of the femoral body (A, A1) in the normal + HUMSCs group. Perforated trabecular bone was found in the distal femoral condyle in the OVX + HUMSCs group (B). An area similar to the structure of the trabecular bone was found in the bone marrow cavity at the injection site of HUMSCs (B1 and B2). A darker trabeculae-like substance was observed in the peripheral region, and a lightly stained dense regular connective tissue-like substance was also observed in the center region (* in B1, B2,). Arrows indicate the direction of transplanted needle. Goldner’s trichrome staining and Sirius red staining were applied to evaluate the change in collagen in the trabecular bone of the left distal femur (C, D, E, and F). The results showed the compact orientations of collagen in the epiphysis and metaphysis of the distal femoral condyle in the normal + HUMSCs group (C, C2, E). Moreover, a substantial amount of collagen was found in the bone marrow cavity of femoral body (C1, E). Loosely packed and irregular collagen was observed in the epiphysis and metaphysis of the distal femoral condyle in the OVX + HUMSCs group (D, F). An area similar to the structure of trabecular bone ossification was observed in the bone marrow cavity at the transplantation location of HUMSCs (D1, D2, and D3). A lightly stained dense regular connective tissue-like substance was also observed in the center region of the trabeculae-like substance (* in D1–D3). A prominent increase in the trabecular bone and collagen content in the metaphysis was found in the normal + HUMSCs group compared with those in the normal + phosphate-buffered saline (PBS), OVX + PBS, and OVX + HUMSCs groups (G, H, I). Scale bar: 100 μm. *Versus normal + PBS group, *P* < 0.05. ^#^ Versus normal + HUMSCs group, *P* < 0.05.

H&E staining showed a dense arrangement of trabeculae in the epiphysis and metaphysis and less Tb.Sp around the track of implantation in the normal + HUMSCs group (Figs. 5A, A2). The trabecular content was higher in the normal + HUMSCs group than in the normal + PBS group (Fig. 5G). In addition, a great quantity of trabeculae was observed in the BM cavity of the left femoral body in the normal + HUMSCs group (Fig. 5A1). Only a small amount of trabeculae was observed in both the epiphysis and metaphysis in the OVX + HUMSCs group (Fig. 5B); this amount was significantly lower than that in the normal + PBS and normal + HUMSCs groups but not significantly different from that in the OVX + PBS group (Fig. 5G). A rod-like material similar to the trabecular bone was observed at the location where HUMSCs were transplanted into the BM of the left femur in the OVX + HUMSCs group (Fig. 5B, B1, B2). Further magnification of the image revealed the presence of a trabeculae-like substance in the peripheral region of the rod-like material. However, a dense regular connective-like tissue with closely arranged and lightly stained cells was found in the center of this rod-like material (Fig. 5B-B2).

Goldner’s trichrome staining showed compact arrangement of collagen with less Tb.Sp in the epiphysis and metaphysis of the left distal femur in the normal + HUMSCs group (Fig. 5C, C2). Compared with the normal + PBS group, the collagen content substantially increased in the epiphysis of the left distal femur in the normal + HUMSCs group (Fig. 5H). A considerable number of trabeculae were observed in the BM cavity of the left femoral body in the normal + HUMSCs group (Fig. 5C1). Less collagen in the trabecular bone was observed only in the epiphysis and metaphysis of the left femur in the OVX + HUMSCs group (Fig. 5D). The collagen content in the metaphysis in the OVX + HUMSCs group was significantly lower than that in the normal + PBS and normal + HUMSCs groups but not significantly different from that in the OVX + PBS group (Fig. 5H). A rod-shaped collagen-containing substance was observed in the BM of the left femoral body at the injection site of HUMSCs in the OVX + HUMSCs group (Fig. 5D, D1, D2). A trabecular-like structure, which stained dark and contained lamellae, had already developed around the region of the rod-like material. However, the compact arrangement of cells, which stained light and was found in the center of the rod-like material, had not yet formed the structure of the trabecular bone but formed the structure of a dense regular connective tissue (Fig. 5D1, D2, D3).

Sirius red staining revealed a dense arrangement of collagen in the condyle of the left distal femoral in the normal + HUMSCs group (Fig. 5E). A prominent increase in the collagen content was observed in the normal + HUMSCs group compared with that in the normal + PBS group (Fig. 5I). In addition, a substantial number of trabeculae were found in the condyle and epiphysis of the left femoral body in the normal + HUMSCs group (Fig. 5E). Only few trabeculae were observed in the epiphysis and metaphysis of the left distal femur in the OVX + HUMSCs group (Fig. 5F), which were significantly lower than those in the normal + PBS and normal + HUMSCs groups but not significantly different from those in the OVX + PBS group (Fig. 5H). A red, rod-shaped, collagen-like substance was observed at the HUMSC injection site in the BM cavity of the left femoral body in the OVX + HUMSCs group (Fig. 5F).

### Transplantation of HUMSCs Reduced the Number of Osteoclasts

**Fig. 6.**
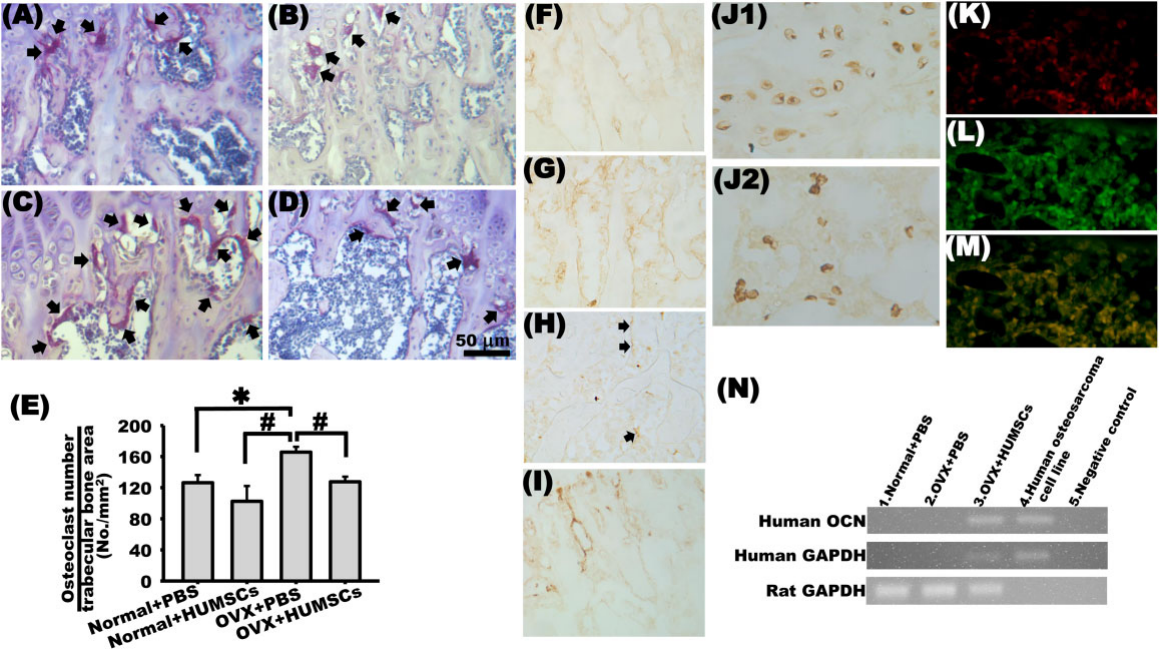
Engrafted human umbilical cord-derived mesenchymal stem cells (HUMSCs) differentiate into osteoblasts and inhibit the number of osteoclasts at the distal femur. TRAP staining was performed to detect osteoclasts (arrows) in the metaphysis of the distal femur in the normal + phosphate-buffered saline (PBS; A), normal + HUMSCs (B), ovariectomy (OVX) + PBS (C), and OVX + HUMSCs (D) groups. The number of osteoclasts per trabecular bone area (no./mm^2^) was quantified. The results indicated no difference in the osteoclast number among the normal + PBS, normal + HUMSCs, and OVX + HUMSCs groups. However, the osteoclast number was significantly higher in the OVX + PBS group than in the other 3 groups (E). Histological sections were immunologically stained with anti-osteocalcin antibody to identify osteocalcin expression near the trabeculae in the metaphysis in each group. The results revealed considerably osteocalcin expression around trabeculae in the normal + HUMSCs group (G). Some osteocalcin expression was detected in the normal + PBS and OVX + HUMSCs groups (F and I). In the OVX + PBS group, only slight expression was detected (H, arrows). Histological sections obtained from the left distal femur were stained with antihuman nuclei antibody to label HUMSCs. Human nuclei were found within the bone marrow cavity (J2) and trabeculae (J1). Double immunofluorescence revealed that numerous HUMSCs (rhodamine, red in K) and abundant osterix (fluorescein, green in L) existed in the rat femur. The merged image showed that many HUMSCs did differentiate into osterix-expressing osteoblasts (M). The results of RT-PCR further demonstrated that the grafted HUMSCs expressed human osteocalcin at the messenger RNA level (N). *Versus normal + PBS group, *P* < 0.05. #Versus OVX + PBS group, *P* < 0.05.

To quantify the number of osteoclasts in the metaphysis, tartrate-resistant acid phosphatase (TRAP) staining was performed. The number of osteoclasts within the left femur slightly decreased in the normal + HUMSCs group; however, this number was not significantly different from that in the normal + PBS group (Fig. 6A, B, E). The number of osteoclasts within the left femur significantly increased in the OVX + PBS group compared with that in the normal + PBS and normal + HUMSCs groups (Fig. 6C, E). The number of osteoclasts within the left femur in the OVX + HUMSCs group was not significantly different from those in the normal + PBS and normal + HUMSCs groups but was considerably lower than that in the OVX + PBS group (Fig. 6D, E), indicating that HUMSCs could inhibit the differentiation or cellular activity of osteoclasts.

### Transplantation of HUMSCs Could Promote Synthesis of Osteocalcin in Osteoblasts

To label and quantify the osteocalcin of osteoblasts in the femur, rabbit antiosteocalcin antibody was used. The normal + PBS group exhibited distinct osteocalcin expression around the trabeculae in the femoral metaphysis (Fig. 6F). An increased level of osteocalcin was also observed in the peripheral region of the trabeculae in the left femur in the normal + HUMSCs group (Fig. 6G). The osteocalcin expression level was not evident around the trabeculae in the OVX + PBS group (Fig. 6H). However, the osteocalcin level was higher in the OVX + HUMSCs group than in the OVX + PBS group (Fig. 6I), suggesting that HUMSC transplantation promoted the synthesis of osteocalcin by osteoblasts.

### HUMSCs Survive and Differentiate into Osteoblasts in the Rat BM

We used antihuman-specific nuclei antigen to label and identify the nuclei of human cells, which showed that HUMSCs survived in the BM cavity, were present in the lamellae, and formed trabecular bone-like tissues 2 mo after transplantation (Fig. 6J1). We suggest that engrafted HUMSCs differentiated into osteoblasts. However, some HUMSCs were found within the BM cavity instead of the trabeculae (Fig. 6J2). To further confirm whether HUMSCs had differentiated into osteoblasts in rat femur, human osteocalcin was detected by RT-PCR. Human osteosarcoma cell line acted as a positive control. As shown in Fig. 6N, against the positive detection of the human osteosarcoma cell line, human osteocalcin messenger RNA was nondetectable in the normal + PBS and OVX + PBS groups. Notably, human osteocalcin was detected in the femur of the OVX + HUMSCs group. The double immunostaining was performed with antihuman-specific nuclear antigen and anti-osterix antibodies for the differentiation of HUMSCs (Fig. 6K–M). We found that numerous HUMSCs existed in the femur of OVX + HUMSCs group, at the same time, abundant osterix were located in the cytoplasm of osteoblasts and the margins of trabeculae (Fig. 6K, L). The majority of engrafted HUMSCs were expressed osterix (Fig. 6M).

### In Vitro Coculture System Indicates that HUMSCs Do Not Trigger Osteoblasts to Differentiate into Mature Osteocytes

**Fig. 7.**
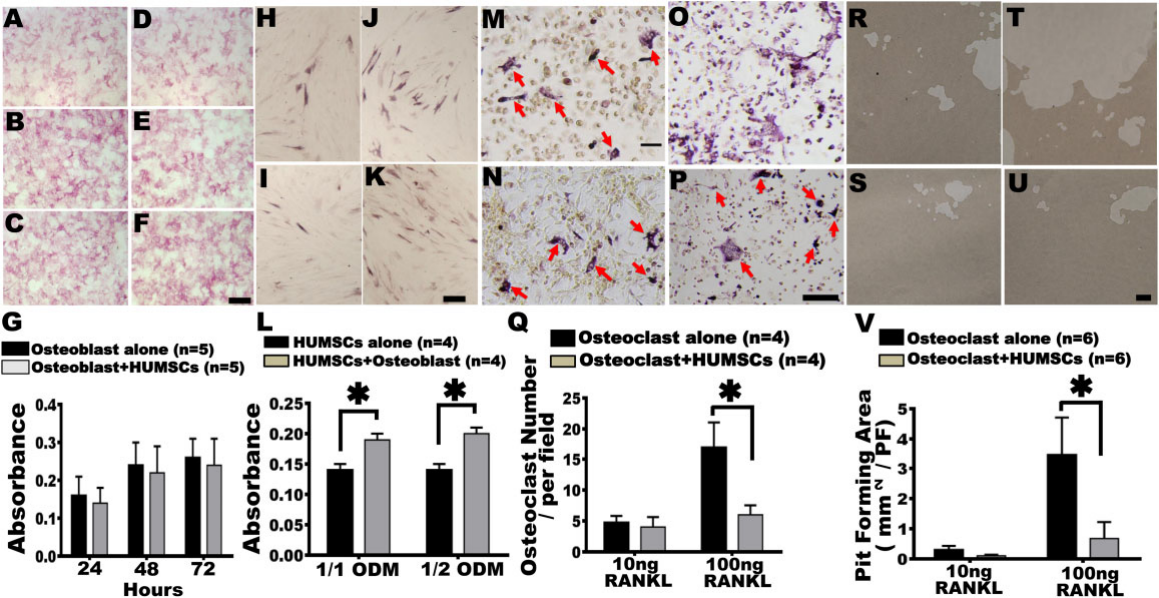
In vitro coculture system demonstrate that osteoblasts promoted differentiation of human umbilical mesenchymal stem cells (HUMSCs) into mature osteocytes and HUMSCs inhibited osteoclast progenitor cells from differentiating into osteoclasts. To investigate the effect of HUMSC intracellular alkaline phosphatase expression on osteoblasts, an in vitro coculture system of HUMSCs and osteoblasts was applied. Osteoblasts (2 × 10^5^ cells) were cultured in the presence (D–F) or absence (A–C) of HUMSCs in serum-free Dulbecco’s modified Eagle’s medium containing 2% bovine serum albumin for 24 h (A, D), 48 h (B, E), and 72 h (C, F). No significant difference was found in the expression of intracellular alkaline phosphatase between osteoblasts alone and osteoblast cocultured with HUMSCs (G). To elucidate whether osteoblasts influence the differentiation of HUMSCs into osteocytes, an in vitro coculture system of HUMSCs and osteoblasts was applied. A 1/1 or 1/2 osteoblastic differentiation medium (ODM) was administered to induce the differentiation of HUMSCs into osteocytes. After 14 d of treatment, the fraction of cells expressing alkaline phosphatase was quantified in single-culture HUMSCs in 1/1 ODM (H) or 1/2 ODM (I) and in coculture osteoblast + HUMSCs in 1/1 ODM (J) or 1/2 ODM (K). The results indicated HUMSCs cocultured with osteoblasts in either 1/1 or 1/2 ODM showed a higher alkaline phosphatase expression level (L). To investigate the effect of HUMSCs on the cellular activity and the number of osteoclasts, an in vitro coculture system of HUMSCs and osteoclasts was applied. The bone marrow cells (10^6^ cells) were cultured in the presence (N and P) or absence (M and O) of HUMSCs (3 × 10^3^ cells) with 10 (M and N) or 100 (O and P) ng RANKL treatment for 9 d to induce osteoclast differentiation. After treatment with 100 ng RANKL, the number of differentiated osteoclasts significantly increased in the single-culture system but significantly decreased in the coculture system (osteoclast + HUMSCs) (Q). Arrows indicate osteoclasts. Rat bone marrow cells were seeded onto an osteoassay plate and were cultured with (S and U) or without (R and T) HUMSCs (3×10^3^ cells) under treatment with 10 (R and S) or 100 (T and U) ng RANKL for 9 d to trigger osteoclast differentiation. The resorbed bone areas were then measured quantitatively to evaluate the activity of osteoclasts. Under 100 ng RANKL induction, the osteoclast + HUMSCs group showed a prominent decrease in the pit formation area (V). Scale bar: 100 μm. *HUMSCs alone versus HUMSCs + osteoblast, *P* < 0.05. *Osteoclast alone versus osteoclast + HUMSCs at 100 ng RANKL treatment, *P* < 0.05.

To investigate whether HUMSCs promote the differentiation of osteoblasts into mature osteocytes, osteoblasts were cultured alone in vitro or with HUMSCs. After 24, 48, and 72 h, no significant difference in the alkaline phosphatase expression level was found between osteoblasts cocultured with HUMSCs and osteoblasts cultured alone (Fig. 7A–G).

### In Vitro Coculture System Revealed that Osteoblasts Promoted Differentiation of HUMSCs into Mature Osteocytes

To examine whether osteoblasts induce differentiation of HUMSCs into mature osteocytes, HUMSCs were cultured alone or with osteoblasts in vitro for 2 wk. The expression level of alkaline phosphatase was evaluated under the formula of 1/1 or 1/2 osteogenic differentiation media. In the single-culture system, alkaline phosphatase was expressed in some HUMSCs under the treatment of 1/1 or 1/2 osteogenic differentiation medium (Fig. 7H–I, L). The percentages of HUMSCs expressing alkaline phosphatase increased when osteoblasts were cocultured with HUMSCs (*P* < 0.05; Fig. 7J–L).

### Coculturing with HUMSCs Significantly Reduced the Number and Activity of Osteoclasts Differentiated from Osteoclast Progenitor Cells

To investigate whether HUMSCs affect osteoclast differentiation, osteoclast progenitor cells were cultured with or without HUMSCs and subsequently treated with RANKL for 9 d to induce differentiation into osteoclasts. Osteoclasts were identified using an acid phosphatase leukocyte kit. After treatment with 10 ng RANKL, no significant difference was observed between single- or cocultured osteoclast progenitor cells (Fig. 7M, N, Q). Furthermore, after treatment with 100 ng RANKL, the number of single-cultured osteoclast progenitor cells that differentiated into osteoclasts was 16.95 ± 4.03 per field. By contrast, only 6.02 ± 1.56 osteoclasts per field differentiated from osteoclast progenitor cells cocultured with HUMSCs, which is significantly lower than that in single-cultured osteoclast progenitor cells (Fig. 7O, P, Q). These results suggested that HUMSCs inhibited osteoclast progenitor cells from differentiating into osteoclasts.

To examine whether HUMSCs influence the activities of osteoclasts, an osteoclast resorption assay was conducted. Briefly, osteoclast progenitor cells were seeded onto a 24-well osteoassay plate coated with inorganic crystalline calcium phosphate in the presence or absence of HUMSCs. After treatment with 10 or 100 ng RANKL for 9 d, cells were induced to differentiate into osteoclasts, and bone resorption activity was quantified. The osteoclasts treated with 10 ng RANKL had a resorbed area of 0.30 ± 0.13 mm^2^ per field when cultured alone, which was not significantly different from that of those cocultured with HUMSCs (0.10 ± 0.04 mm^2^ per field; Fig. 7R, S, V). After treatment with 100 ng RANKL, osteoclast progenitor cells were cultured with or without HUMSCs. The resorbed areas were 3.46 ± 1.24 and 0.68 ± 0.55 mm^2^ per field in the single-cultured osteoclast progenitor cells and osteoclast progenitor cells cocultured with HUMSCs, respectively (Fig. 7T, U, V). These results indicated that HUMSCs could effectively inhibit the bone resorption activity of osteoclasts.

## Discussion

### Transplanted HUMSCs Differentiate into Osteoblasts and Promote Osteogenesis

Studies have reported that stem cells originating from sources other than Wharton’s jelly of the umbilical cord can ameliorate osteogenesis. Ye et al. isolated and purified subcutaneous adipose-derived stem cells from rabbits 8 mo after OVX. After differentiation induction in osteogenic differentiation medium, 5 × 10^6^ osteogenically differentiated adipose stem cells were transplanted into the left femoral condyle. After 12 wk, the bone density considerably improved at the transplantation location^18^.

Furthermore, Aggarwal et al. induced the differentiation of human CD34-positive umbilical cord blood stem cells into osteogenic lineages. They subsequently injected 5 × 10^5^ differentiated umbilical cord blood stem cells into the ventricles of NOD/SCID mice with corticosteroid-triggered osteoporosis. After 28 d, the femoral trabeculae and serum osteocalcin level increased significantly^19^. Previous studies demonstrated that engrafted stem cells might provide paracrine effects to stimulate proliferation and osteogenic differentiation of recipient bone marrow-derived MSCs (BMMSCs) and enhance bone regeneration in vitro and in vivo.

Studies have also treated osteoporosis by using BMMSCs. Takada et al. used senescence-accelerated mouse prone 6 (SAM/P6) transgenic mice as an animal model. Without any treatment, osteoporosis occurred in SAM/P6 mice at the age of 12 mo. The transplantation of BM cells into the distal femurs of SAM/P6 mice showed amelioration of osteoporosis 12 mo later^20^. Sui et al. also demonstrated that allogeneic MSC therapy promotes and prevents glucocorticoid-induced osteoporosis in rats^21^. Similar results were reported in multiple myeloma induced lytic bone disease, whereby intrabone injection of BMMSCs promoted bone formation^22^. However, Huang et al. reported results contradictory to those of the aforementioned studies. In bilateral OVX rats, BMMSCs were transplanted 10, 45, and 90 d after surgery. On the 135th-day postsurgery, the systemic administration of allogeneic MSCs from the same donor had no obvious effect on osteoporotic bone loss in OVX rats. However, the repeated injection of allogeneic BMMSCs from different donors might promote bone loss in OVX rats^23^.

The present study demonstrated that there are two therapeutic mechanisms of the engrafted HUMSCs. One is differentiation into osteoblasts to improve bone deposition, and the other is through release of cytokines to inhibit the activation of osteoclasts. The trabecular network showed a dense and regular orientation from the distal femoral epiphysis, metaphysis, to femoral body in the normal + HUMSCs group, suggesting that the microenvironment of the normal BM accelerates the remodeling and differentiation of stem cells into osteocytes. However, 2 mo after transplantation, rats in the OVX + HUMSCs group demonstrated tightly packed connective tissues in the femoral body at the injection site of 2.5 × 10^6^ HUMSCs. The outer layer of this connective tissue possessed structures similar to the trabeculae, indicating that the pathological environment of the BM in OVX rats may result in the slower differentiation of HUMSCs into osteoblasts and bone mass reconstruction, in contrast to that in the normal + HUMSCs group. Micro-CT images obtained showed no significant improvements in the trabecular bone in the epiphysis and metaphysis in the OVX + HUMSCs group. However, immunohistochemical staining revealed the upregulation of osteocalcin in the metaphysis. This finding suggests that the location examined was far from the injection site of HUMSCs or that more time was needed for osteogenesis to occur.

### HUMSCs Cocultured with Rat Osteoblasts Showed Increased Differentiation into Osteogenic Lineages

Tong et al. induced the differentiation of human BMMSCs into osteocytes and subsequently cocultured them with human embryonic stem cells by using the Transwell System. The Transwell System allows no direct contact between osteocytes and embryonic stem cells but renders the exchange of medium once. Twenty-eight days later, the deposition of calcium and phosphate was observed in embryonic stem cells through Von Kossa and Alizarin Red staining, respectively. At the same time, the synthesis and secretion of osteocalcin were also detected^24^. Similarly, Ye et al. isolated and purified subcutaneous adipose-derived stem cells from rabbits and differentiated them into osteoblasts by using osteogenic differentiation medium. Subsequently, osteogenically differentiated adipose stem cell–conditioned medium was applied for culturing BMMSCs. It was found that the fraction of osteoblasts derived from BMMSCs increased significantly^18^. In addition, HUMSC expression of alkaline phosphatase increased when cocultured with rat osteoblasts; this finding is consistent with that of our study. The BM microenvironment induces HUMSC differentiation into osteoblasts and osteocytes or modulates the differentiation rates.

### HUMSCs Could Reduce the Differentiation Capability of Osteoclast Progenitor Cells into Osteoclasts

Oshita et al. cocultured human BMMSCs with peripheral blood mononuclear cells in osteoclast induction medium by using the Transwell System. The capability of differentiating into osteoclasts was then quantified by coculturing peripheral blood mononuclear cells with BMMSCs. The results indicated that the number of osteoclasts differentiated from peripheral blood mononuclear cells cocultured with BM stem cells decreased. In addition, the osteoclast resorption ability decreased, suggesting that human BMMSCs inhibited osteoclast activities^25^. Similar results were reported by Li and colleagues who also demonstrated that coculturing of osteoclast precursors with BMMSCs in osteoclast medium resulted in fewer mature multinucleated osteoclasts than when they were cultured alone^22^. Although different stem cells were used, similar results were obtained in the present study. When cocultured with rat osteoclast progenitor cells in osteoclast differentiation medium, HUMSCs significantly reduced the number and activity of osteoclasts even under a high concentration of RANKL.

## Conclusion

In this study, a considerable number of xenotransplanted HUMSCs differentiated into osteoblasts and concurrently released cytokines to impaired osteoclast activity in the recipient BM cavity. Our findings provide a new therapeutic strategy and demonstrate that HUMSCs can clinically resolve bone-related medical conditions such as osteoporosis and bone fractures.

## Supplemental Material

Supplemental Material, Pages_from_Figure_(CT-1813_revised) - Xenograft of Human Umbilical Mesenchymal Stem Cells from Wharton’s Jelly Differentiating into Osteocytes and Reducing Osteoclast Activity Reverses Osteoporosis in Ovariectomized Rats Click here for additional data file. Supplemental Material, Pages_from_Figure_(CT-1813_revised) for Xenograft of Human Umbilical Mesenchymal Stem Cells from Wharton’s Jelly Differentiating into Osteocytes and Reducing Osteoclast Activity Reverses Osteoporosis in Ovariectomized Rats by Yu-Show Fu, Chia-Hui Lu, Kuo-An Chu, Chang-Ching Yeh, Tung-Lin Chiang, Tsui-Ling Ko, Mei-Miao Chiu, and Cheng-Fong Chen in Cell Transplantation
