# Supplementary material for: Xenograft of Human Umbilical Mesenchymal Stem Cells from Wharton’s Jelly Differentiating into Osteocytes and Reducing Osteoclast Activity Reverses Osteoporosis in Ovariectomized Rats
Source: Cell Transplant. 2018 Mar 22;27(1):194–208. doi: 10.1177/0963689717750666 (PMC6434481; doi:10.1177/0963689717750666)

Supplemental Figure 1

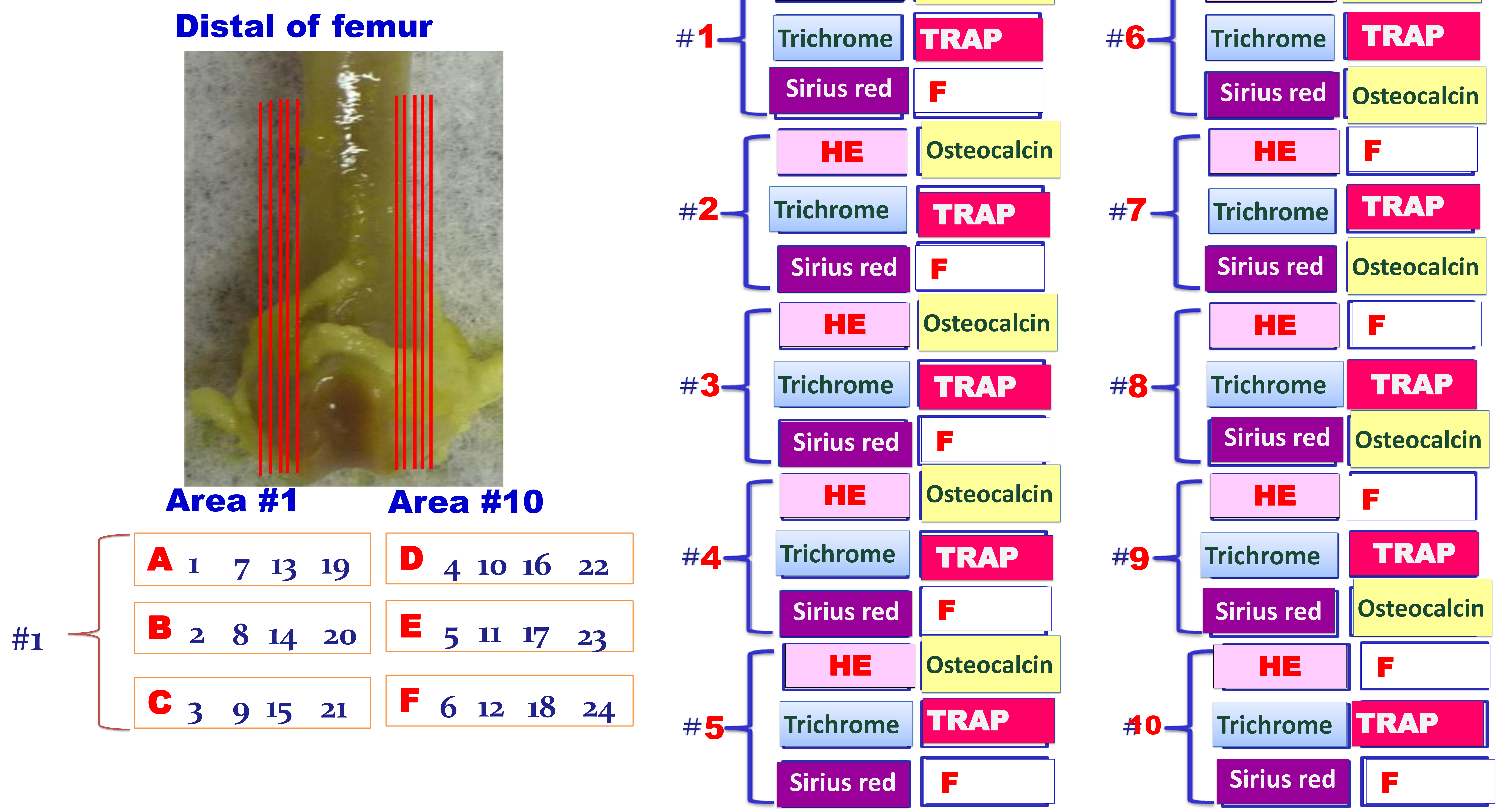

Supplemental Figure 2

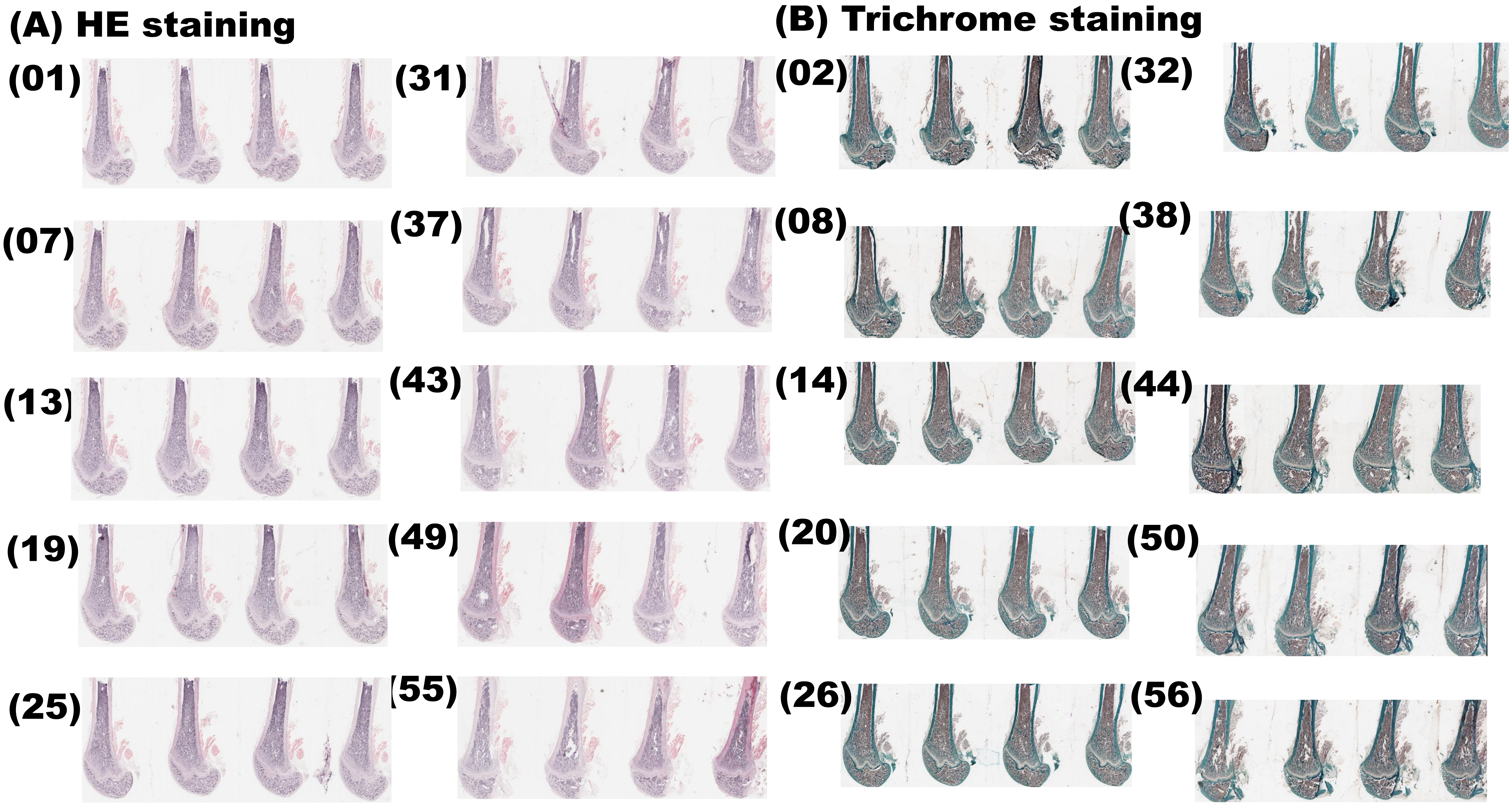

Supplemental Figure 3

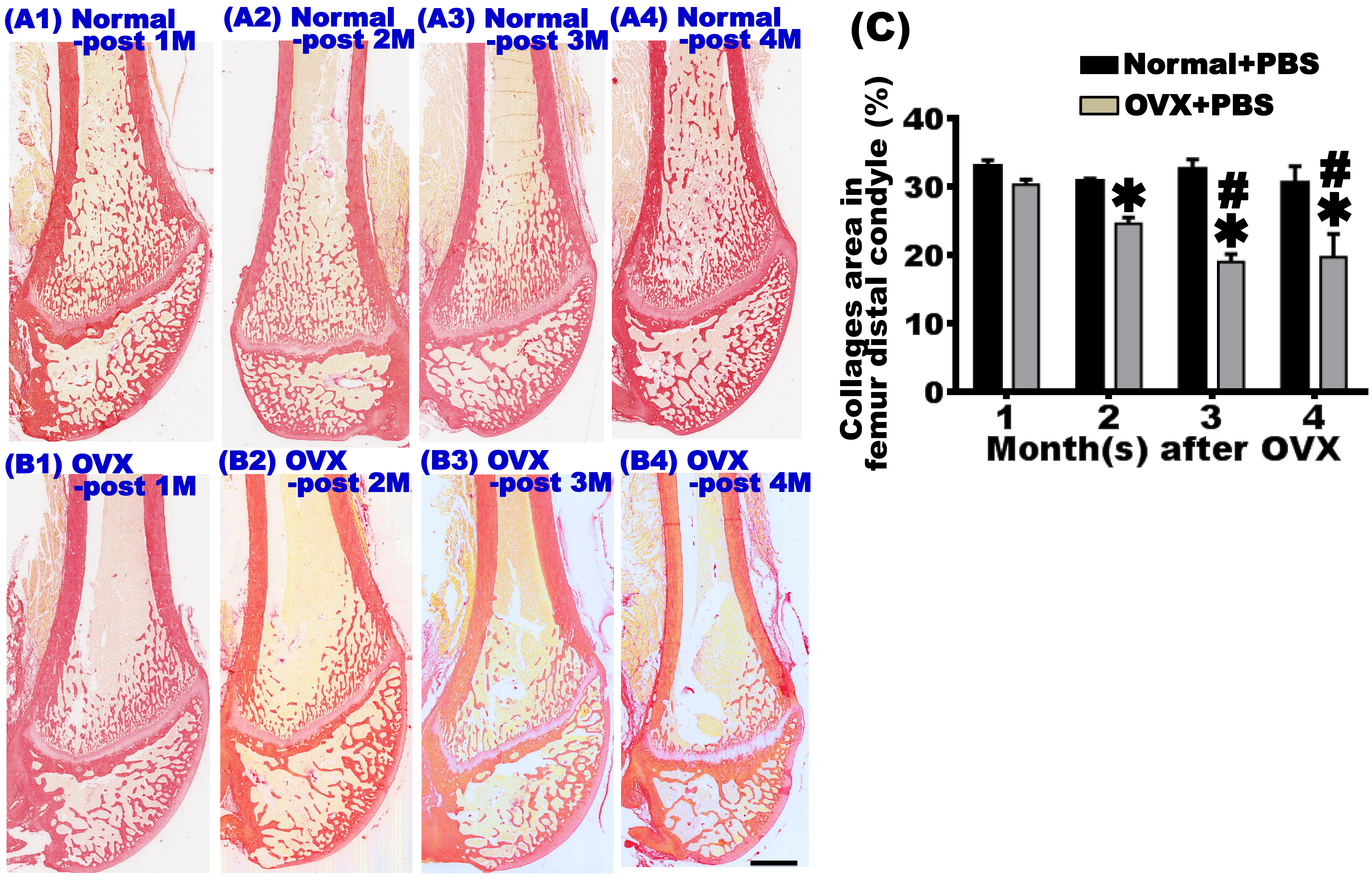

Supplement: Supplemental Material, Pages_from_Figure_(CT-1813_revised) - Xenograft of Human Umbilical Mesenchymal Stem Cells from Wharton’s Jelly Differentiating into Osteocytes and Reducing Osteoclast Activity Reverses Osteoporosis in Ovariectomized Rats [file Pages_from_Figure_(CT-1813_revised).pdf]
